# Modelling representations in speech normalization of prosodic cues

**DOI:** 10.1038/s41598-022-18838-w

**Published:** 2022-08-27

**Authors:** Chen Si, Caicai Zhang, Puiyin Lau, Yike Yang, Bei Li

**Affiliations:** 1 Department of Chinese and Bilingual Studies, The Hong Kong Polytechnic University, Kowloon, Hong Kong SAR China; 2 Hong Kong Polytechnic University-Peking University Research Centre on Chinese Linguistics, Kowloon, Hong Kong SAR China; 3 Research Centre for Language, Cognition, and Neuroscience, University of Hong Kong, Pok Fu Lam, Hong Kong SAR China; 4 Department of Statistics and Actuarial Science, University of Hong Kong, Pok Fu Lam, Hong Kong SAR China; 5 Department of Chinese Language and Literature, Hong Kong Shue Yan University, North Point, Hong Kong SAR China

**Keywords:** Human behaviour, Learning and memory

## Abstract

The lack of invariance problem in speech perception refers to a fundamental problem of how listeners deal with differences of speech sounds produced by various speakers. The current study is the first to test the contributions of mentally stored distributional information in normalization of prosodic cues. This study starts out by modelling distributions of acoustic cues from a speech corpus. We proceeded to conduct three experiments using both naturally produced lexical tones with estimated distributions and manipulated lexical tones with f0 values generated from simulated distributions. State of the art statistical techniques have been used to examine the effects of distribution parameters in normalization and identification curves with respect to each parameter. Based on the significant effects of distribution parameters, we proposed a probabilistic parametric representation (PPR), integrating knowledge from previously established distributions of speakers with their indexical information. PPR is still accessed during speech perception even when contextual information is present. We also discussed the procedure of normalization of speech signals produced by unfamiliar talker with and without contexts and the access of long-term stored representations.

## Introduction

The lack of invariance problem is a fundamental problem in speech perception research, which refers to the fact that listeners need to overcome the acoustic differences of speech sounds produced by multiple speakers^1^. Early studies attempted to tackle the problem by proposing acoustic invariance, which aims to find invariant acoustic cues produced by all speakers^2,3^. For example, it is believed that the shape of spectrum serves as a cue for place of articulation. However, those invariant cues have not yet been found for vowels and fricatives since their production varies to a great extent depending on the size and shape of vocal tract (See^4^ for a review). Later, the concept of normalization was proposed to solve this issue, which refers to a perceptual process that normalizes talkers’ variability^5–9^. This approach is based on the idea that speech signals vary among talkers, and perceptual normalization can offer relational invariance. For example, the perception of VOT (voice onset time) can be adjusted to the speaking rate of a talker^10,11^.

Moreover, listeners may establish mental representations of speech sounds, and judge the incoming signal based on their mental representations. Regarding the mental representation, there is a debate over the abstract and episodic nature of phonological representation. The abstractionist theories of phonology propose minimized underlying representations with no extraneous details^12^. The phonological theories successfully explain how speaker encode phonological information in their mind and how they acquire generalizations while learning new words^13^. Abstract representation is needed to explain the ability of generating and processing new forms. Evidence from neurolinguistic studies also supported abstraction in that the neural representation was found to be categorical in the posterior superior temporal gyrus^14^.

On the other hand, episodic theories of phonology involve more social and contextual variations^15^ compared to abstractionist theories of phonology. Human beings are capable of forming mental representations of events with great details and also recalling the detailed representations^16^. For example, early experiments showed that infants can include phonetic information and indexical properties in representing sounds^17^. It is thus likely that mental representations can be detailed with indexical properties.

Considerable computation is required in speech perception due to variability in the acoustic signals of speech sounds produced by multiple speakers. For example, it is reported that in fricative production, one speaker’s production of the consonant /s/ may sound similar to another speaker’s /ʃ/ sound^18^. The observed talker differences are usually conditioned by factors including individual variations of the vocal tract such as length and size of the vocal tract as well as indexical variables such as gender and age. For example, because the length of vocal tract of men tend to be longer than women, vowel production can differ between female and male speakers, where formants tend to be lower for male speakers^5^.

The statistical distributions of acoustic cues for each speaker can be different, which makes the perception of speech signals challenging since listeners need to map the same physical values to different linguistic categories^19^. Therefore, listeners need to undergo talker normalization, which maps speech signals to linguistic categories. One way of normalizing speech signals is through category-intrinsic procedures, where the intrinsic acoustic information from a certain category is used solely to normalize speech tokens^8,20–23^. For example, in vowel normalization, the interval of formants can be calculated to reveal relational invariance independent of the absolute formant values^24^ or f0 may offer a reference point for normalizing vowel production by multiple speakers^8^. This approach has the basic assumption that the variability of speech signals can be considered as a useful and informative source for speech perception^25^.

In addition, exemplar theories have been applied to speech perception and lexical representations, which also favour the position that the representation of speech sounds and even lexical items are episodic, and entails exemplars with acoustic details of speaker voices^7,26–30^.

Ideally, we do need a theory of representations that may explain how both abstract and episodic representations might co-exist in individuals’ representations of speech sounds.

### Parametric representations

Parametric representations may help link the above-mentioned two theories. Parametric representations of phonetic distributions have been proposed to explain how phonetic details impact representations in memory and the fact that phonological encoding is associated with parametric information of phonetic details^31^. For example, in the word “notice”, the phoneme /t/ is realized as /ɾ/. The phoneme /r/ is produced as /ɹ/ in “worries”. The main differences between /ɾ/ and /ɹ/ lie in the values of F3. If their distributions of F3 values are not overlapping, then the phonetic values can be mapped onto two distinct phonemes easily. However, the phonetic distributions of F3 values of the two allophones are overlapped and are also subject to gender variations. Therefore, the decision of the phonemes can be dependent on the indexical information of speakers. From the listeners’ perspective, it is also argued that listeners can form parametric representations of phonetic distributions and they may update the parameters of phonetic distributions through learning^31^.

It is reported that learners are aware of distributional statistics in speech perception^13,32,33^. For example, it is reported that listeners were sensitive to the long-term regularities of the language in speech perception of f0 and VOT, and they could also flexibly adjust the acoustic dimensions to talker idiosyncrasies^13^. Also, Theodore and Monto tested whether the representations built for a certain talker reflect long-term or only short-term exposure to that talker using consonants with manipulated distributions^33^. They found out that listeners were able to use accumulated information in their long-term representation as well as making adjustment to short-term exposure. In addition, listeners may also exploit indexical information of the talker in mapping speech signals to linguistic categories. Listeners may also adjust their speech perception according to gender or age^29^, indicating that they may have stored parametric representations with respect to certain socio-indexical group and the representations may be accessed during speech perception.

Although parametric representations have been proposed, the characteristics of such parametric representations have not been clearly defined. Moreover, few experiments have been conducted to further our understanding of parametric representations. It remains to be explored why highly detailed memories and abstractness can exist simultaneously in our mental representations and how they are accessed in the normalization of speech, especially for tone perception. Although there is an implicit assumption that a listener’s past experience shapes the representations of speech sound categories in the listener’s mind, few studies have measured population acoustic distributions from a large sample of speech materials and designed perception experiment based on estimated population distributions. It is therefore important for us to design experiments to test how parametric representations are employed in speech perception and what the characteristics are of parametric representations.

### Speech perception of Cantonese tones

**Figure 1:**
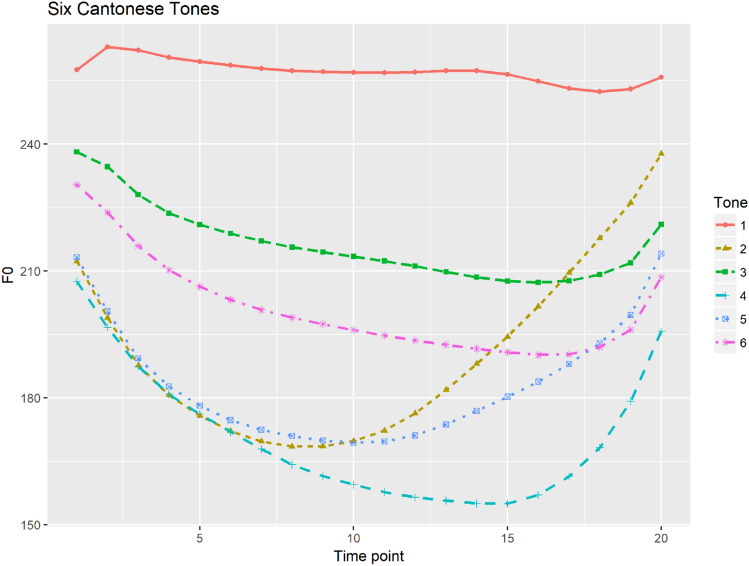
Six Cantonese tones with time-normalized mean f0 contours calculated speech production by seven female speakers.

Cantonese tonal system is relatively complex with six tones (T1 (55/53); T2 (25); T3 (33); T4 (21); T5 (23) and T6 (22)) in open syllables and three additional tones on the checked syllables ending in a consonant, considered as the three counterparts of the three level tones on open syllables^34,35^. F0 is the primary acoustic cue in Cantonese tone perception both in real speech^36^ and synthesized speech^37^. Figure 1 shows six Cantonese tones with time-normalized tonal contours produced by seven female speakers.

**Figure 2:**
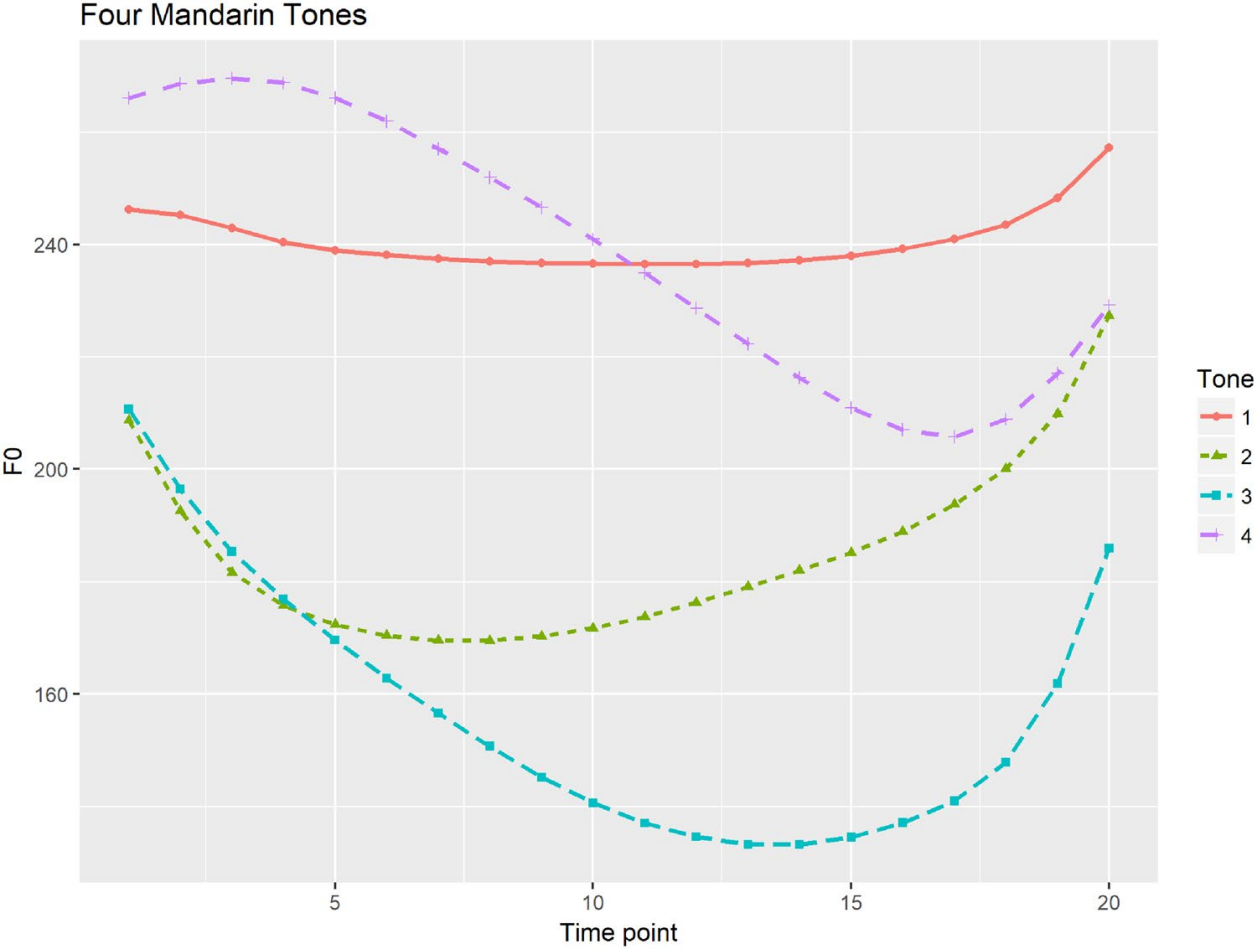
Four time-normalized Mandarin tones based on the mean f0 values calculated from seven female speakers.

Since Mandarin has a simpler tonal system with four tones showing more distinct tonal contours compared to Cantonese, Mandarin tones produced by multiple talkers can be well categorized and thus talker variability only had limited influence in tone perception^30^. Figure 2 shows four Mandarin tones with time-normalized mean f0 contours produced by seven female speakers. However, it is reported that perception of Cantonese tones relied more on distinctions of f0 height and thus were more subject to talker variabilities^38^. Since Cantonese tones have more acoustic overlap among multiple talkers^39^, talker variability tend to greatly affect tone perception, especially for tones with similar tonal contours such as level tones in Cantonese^40^. Similarly, categorical perception of Cantonese level tones tend to be weaker. Francis and his colleagues compared identification and discrimination of a low-level to high-level, a rising-to-level tonal continua and a low falling to high rising continua among native Cantonese listeners^41^. Consistent with^42^’s findings, level tones were not perceived categorically by native Cantonese listeners; while category boundaries were present in the identification task, no discrimination peaks were found at the category boundary and identification response did not predict discrimination level across the continuum. However, results of the rising-to-level contour were consistent with those reported in^16^. For this continuum, Cantonese speakers showed clear discrimination peaks at the category boundaries and discrimination proportions were predicted by the results of the identification task. These results of weak categorical perception of level tones raise the question of how native speakers identify level tones especially when the talker information and contexts are deprived.

From previous research on talker normalization, three level Cantonese tones have received extensive attention^41,43–45^. The three level tones (high tone T1(55/53), mid tone T3(33) and low tone T6(22)) offers us a great chance to test the knowledge of f0 distributions because (1) the three tones can be distinguished primarily by a single phonetic dimension, namely pitch height, and other acoustic cues such as duration and intensity are similar among the tones, which makes it easy to control in the experiment^44^; (2) it is more feasible to estimate the parameters of f0 distributions because of the simple level tonal shape and thus easy to simulate f0 values from the estimated distributions. The current study is a first step to test our hypothesis that the representations are shaped by statistical distributions. The results we generate from experiments using a simple phonetic dimension may serve as a base for future studies, where we can further generalize the results to other more complex phonetic cues and multi-dimensional representations.

### Speech normalization

Past research showed that both facial features^46^ and variability in pitch trajectories^47^ contribute to social judgements. Although it is known that listeners excel at forming social representations based on the statistical distributions of facial features and the variability in pitch, less is known about how they use mentally stored statistical distributions and indexical information related to speakers in speech perception.

Talker normalization of speech signals produced by multiple speakers may lead to extra processing costs compared to processing of speech signals by a single speaker^6,7,9^. The Computing Cues Relative to Expectations (C-CuRE) model is proposed for normalization and categorization^32^. In that model, acoustic cues are coded based on their differences from expected values in categorization of cues. For example, fundamental frequency (f0) may serve as a cue to voicing in consonants since higher f0 usually indicates voiceless consonants and lower f0 voiced consonants. Once the talker is identified, speakers may use the information about the talker such as average f0 to judge if the incoming f0 is higher or lower than the average. If the f0 input is higher than the expected average value, then it is to be identified as a voiceless segment. Similarly, it is found that listeners performed better in speech processing if the signals are produced by familiar talkers than those produced by unfamiliar talkers^48,49^. More interestingly, speakers may still handle normalization even if the talker’s identity and acoustic information are not fully available^44^. Learners need to acquire the patterns of variations in the speech signals to effectively process speech from unfamiliar talkers and this perceptual learning process relies heavily on listeners’ past experience and knowledge^50^. Therefore, we believe that an exploration into the characteristics of mentally stored statistical distributions and how the statistical distributions are accessed may help us gain a fuller picture of the normalization process. Most of these current studies and models are based on segmental features, and speech normalization of prosodic cues still remains to be further explored.

In addition, numerous studies explored the adjustment of speech perception due to contextual information such as speech rate, which influences perception of vowel duration^51,52^. This means that normalization may occur using category-extrinsic cues (for a review, see^19,40^), involving acoustic information from an external frame of reference such as the preceding context. Category-extrinsic cues have been shown to be effectively used in normalization of lexical tones. For example, Wong and Diehl showed the extrinsic effects on the target mid tone by shifting the f0 of the preceding sentence two semitones upward, which caused Cantonese listeners to identify the mid tone target as a low tone, whereas a shift downward by three semitones caused the target to be heard as a high tone^43^. Francis et al. tested the degree of shift, and concluded that a two-semitone shift was enough to elicit the expected response: raised contexts for identification of a mid tone as a low tone, and lowered contexts for it to be perceived as a high tone^41^. The normalization effect may be partly subject to the general auditory contrast enhancement mechanism ^53^. The mechanism proposed that contextual information (speech or non-speech) such as the average spectrum may affect perception of new speech stimuli^54^. Other studies showed reduced effects of non-speech contexts and thus speech-specific gestural account has been proposed in addition to general auditory processes^55^. More recent studies proposed that phonological cues may be more important in tone normalization and the extrinsic normalization process may integrate processing of general auditory cues, phonological cues and even other cues such as semantic cues^40,45^. However, the relationship between the mentally stored long-term representations and immediate contextual effects is rarely examined. Whether mentally stored tonal distributions and contextual information compete with each other during normalization still remains to be investigated.

## The current study

The current study specifically tests human listeners’ knowledge about statistical distributions of prosodic cues. The processing of prosodic cues is crucial for perception research since prosodic cues have various linguistic functions such as assisting children and adults in word detection^67^, extracting grammatical patterns and linguistic rules^56–58^. However, research on how distributions of prosodic cues are accessed in speech normalization is still lacking.

We designed speech perception experiments to specifically test how parameters of distributions may contribute to tone processing and interact with other factors. These experiments involve perception of natural and resynthesized speech stimuli by Cantonese native speakers. We propose to answer three research questions: (1) What kind of representations were built and accessed in the normalization of speech by native Cantonese speakers? (2) Does deviation from the estimated population distribution affect the decision by Cantonese speakers? (3) Do mentally stored tonal distributions still play a role when contextual information is present during normalization by Cantonese speakers? To answer these questions, we designed three experiments, which were conducted on native Cantonese speakers: (1) speech perception of real stimuli produced by multiple speakers with estimation of the tonal distribution of each speaker; (2) speech perception of manipulated stimuli simulated from distributions with varied parameters from estimated population distribution; (3) speech perception with simulated contextual information.

Experiment 1 was designed to test the parametric nature of phonetic representations of individual speakers. Using the idea of distributions to fit the observed f0 values, we may have the power to test the underlying patterns for how these f0 values are stored mentally, especially whether listeners have the knowledge about the probability of these f0 values associated with tonal categories. Distributions reflect the probability of f0 values and they are very useful tools to model f0 values. We may use this concept of distributions to test how distributional information contributes to listeners’ perception. Since the three parameters determine the shape of the distributions, significant contributions of the parameters indicate that listeners’ have knowledge about the shape of the distributions and the ability to extract the probability of f0 values in tone perception. If the parameters do not contribute significantly in perception, then listeners have limited ability to extract the probability of f0 values and their mental representations are not parametric. Significant contributions of f0 values in tone perception does not necessarily mean significant contributions of distributional information of f0 as it also includes the probability information of f0 values. In addition, by modelling the distribution of each speaker, we may further test whether listeners have information about the probability of extracted parameters from multiple speakers, reflecting their knowledge of typicality of speakers. It aims to examine identification curves for each parameter to test whether listeners can have access to the probability of extracted parameters from tonal distributions of multiple speakers. If listeners do not have the ability to further calculate the probability of extracted parameters from tonal distributions of multiple speakers, then the identification curve will be relatively flat no matter how the values of each parameter change. Finally, it aims to compare the tonal distributions of tones in speech production and perception. Experiment 2 was designed to test whether native speakers were sensitive to the deviation of parameters from the estimated population distribution parameters using synthesized stimuli. It is a further step to test listeners’ sensitivity to distributional information using synthesized stimuli. Experiment 3 was designed to test if mentally stored distributions still played a role while contextual information was present.

Our study is the first to provide estimation of f0 distributions from a large speech sample of native Cantonese speakers. All the speech perception experiments were designed based on the estimation of population f0 distributions or f0 distributions of individual speakers. We also combined natural and synthesized stimuli in the design of experiments for their complementary strengths to offer a comprehensive test of the parametric representations in normalization of speech. We used sophisticated statistical modelling techniques to test whether parameters of f0 distributions contributed significantly to tone identification, to model speakers’ identification curves, and we compared the f0 distributions based on their perceptual performance to those estimated from speech production. A new procedure of normalization based on probabilistic parametric representations was then proposed to account for the findings.

## Materials and methods

### Pre-screening

Tone merging of Cantonese T3/T6, T2/T5 and T4/T6 has been reported as an on-going process in younger Cantonese speakers, and the merging shows extensive individual differences^31,38^. Some studies found that the potential mergers could distinguish tones with more than 90% accuracy, though lower than the control group^60,61^. However, Fung et al. found that mergers have much lower accuracy in discriminating T4/T6^62^. In order to make sure tone mergers do not affect our results, we pre-screened participants to exclude any potential merger who could not effectively discriminate Cantonese tones.

In the pre-screening procedure, we used the same-different AX discrimination task, presenting a pair of monosyllables in each trial. The pair could consist of either the same stimuli (AA) or different ones (AB/BA). Following designs of previous studies on perception of merging tones^59,61,62^, we included four Cantonese monosyllables /fu/, /ji/, /sɛ/ and /si/ in the pre-screening procedure, which could bear all six Cantonese tones. Four female speakers were recorded reading these four syllables bearing six unchecked tones, and repeated each monosyllable six times. We chose a female native speaker (22 years old with a college degree, without any reported history of speech, hearing, or language difficulties) who could separate six tones clearly, and whose tonal distributions were closest to the estimated population parameters. The average intensity level of all tokens was normalized to 55 dB and the duration of all monosyllables was normalized to 450 ms. In total, there were 60 AB/BA trials (2 syllables /ji///fu/ * 15 combinations * 2 orders) and 24 AA trials (4 syllables * 6 tones). All the tokens were randomly presented using E-prime 2.0.

In this pre-screening procedure, participants were told to judge whether each trial consisted of the same or different tones. The interstimulus interval was set to 500 ms. All twenty-eight native Cantonese speakers attained a mean accuracy rate of 98.00% (SD ± 1.56%) and participated in the perception experiments.

### Experiment 1: natural tone identification in isolation

#### Subjects

Thirty-four native Cantonese speakers (17 females; 17 males; mean age ± sd: 20.07 ± 1.18 yrs) were recruited from the Hong Kong Polytechnic University for speech recording of stimuli with and without contextual information. Another fourteen Cantonese speakers (7 female, 7 male; mean age ± sd: 20.43 ± 1.02 yrs) participated in Experiment 1. All participants were paid to participate in the experiments, and signed informed consent forms in compliance with a protocol approved by the Human Subjects Ethics Sub-committee at the Hong Kong Polytechnic University. All protocols are carried out in accordance with relevant guidelines and regulations. Participants who reported any history of speech, hearing, or language difficulties were not included.

#### Stimuli

The stimuli were recorded using a Shure SM48 microphone in a soundproof booth of the speech lab at the Hong Kong Polytechnic University with a sampling rate of 44.1 k Hz. The recruited 34 native Cantonese speakers (17 female and 17 male) were required to pronounce the two syllables /ji/ and /si/ with the three level tones (T1, T3 and T6) embedded in the carrier sentences for ten times respectively. The carrier sentences for these syllables were 聽聽 /tʰiŋ tʰiŋ / _________ “Listen to_________”. Since the syllable /si/ was treated as a filler syllable, for each repetition, we only segmented the voiced portion of /ji/, and extracted f0 values at 20 normalized time points using the ProsodyPro Praat script^63^. Then we fit a skew-normal distribution to the f0 values of each speaker and extracted three parameters from the distribution of each speaker’s tonal production (location (ξ), scale (ω), and shape (α)) for statistical analyses. We analysed these three parameters because these parameters determine the density function of any skew normal distribution.

To prepare all the target and filler stimuli, we proceeded to extract the syllables /ji/ and /si/ from the carrier sentences. In Experiment 1, f0 values were not manipulated, whereas f0 values of stimuli in Experiment 2 were further manipulated according to simulated distributions. To balance the loudness level of each target stimulus in the two experiments, the average intensity level was normalized to 55 dB and the duration of all syllables were normalized to 450 ms using the software Praat^64^. The syllable /si/ was treated as a filler syllable to ensure that participants were more focused on the experimental tasks. In total, there were 680 stimuli (10 tokens * 34 speakers * 2 syllables).

#### Procedure

Each item from the two sets of stimuli (target and filler stimuli) was presented in isolation (i.e., without a carrier) using the three-alternative forced choice identification task. The two sets of stimuli were presented in isolation using the three-alternative forced choice identification task. We mixed the target and filler words produced by the 34 talkers randomly. All the stimuli were blocked by gender. The order of 34 talker-blocks was also randomized. All the participants wore a headset GMH C 8.100, and they were instructed to choose one monosyllable they heard from a set of three target Cantonese words醫 (/ji55/ “a doctor”), 意 (/ji33/ “meaning”), and 二 (/ji22/ “two”), and the other set of three filler Cantonese words師 (/si55/ “a teacher”), 試 (/si33/ “to test”), and 事 (/si22/ “matter”), by pressing buttons on a computer keyboard. They were given traditional Chinese characters and symbols for tonal contours to choose from upon hearing each stimulus. They were notified about the gender of the speaker.

#### Results

Azzalini described the probability density function (pdf) of a skew-normal distribution with a shape parameter α, i.e., SN (0, 1, α) in the form^65^:

1 $$f\left( x \right) = 2\phi \left( x \right)\Phi \left( {\alpha x} \right)$$

where α is a real number, ϕ(·) is the pdf of a standard normal distribution and Ф(·) is its distribution function. When α = 0, the skew-normal distribution SN(0, 1, 0) reduces to a standard normal distribution N(0, 1). In general, the skew-normal distribution SN(ξ, ω^2^, α) has three parameters, the location ξ, scale ω and shape α. The location parameter determines the location of the distribution, the scale parameter determines the dispersion, and the shape parameter determines the skewness.

We applied a multinomial mixed effects model to investigate whether each parameter estimated from each speaker contributes to tone perception performance of Cantonese speakers. The response tonal category (T1, T3, and T6) was the dependent variable and the parameters extracted from the fitted skew-normal f0 distributions (location, scale, and shape) were the independent variables in the model. Specifically, the dependent variable is the response of the tonal category (T1, T3, and T6) from the listeners who heard trials of T3 produced by 34 Cantonese speakers. Although all the tones produced were T3, the listeners may identify some of them as T1 or T6. For the fixed effect, we modelled the distribution of T3 produced by each of 34 speakers, and the parameters were extracted from the fitted skew-normal f0 distributions (location, scale, and shape). The formulae of the model were listed in the appendix. The analysis was conducted using the R package mixcat ^66^ to fit a multinomial mixed effects model with non-parametric (discrete) distribution for the random effects.

When T6 served as a baseline, the main effects of location (T1 against T6: OR = 1.07, *p* < 0.001; T3 against T6: OR = 1.05, *p* < 0.001) and shape (T1 against T6: OR = 7.77, *p* < 0.001; T3 against T6: OR = 16.28, *p* < 0.001) were positively related to the probability that the tone was identified as T1 or T3 against T6. The main effect of scale did not reach significance. All the two-way and three-way interaction terms reached significance. Similarly, in identifying T1 against T3, the main effect of location (OR = 0.65, *p* = 0.023) and shape (OR = 0.03, *p* < 0.001) were statistically significant in tone identification, but the main effect of scale did not reach significance. All the two-way and three-way interaction terms reached significance except for the interaction between location and scale in identifying Tone 1 against T3.

Essentially, using the multinomial mixed effects model, we showed that listeners have the knowledge about the probability of f0 values associated with tonal categories since all the three parameters contributed significantly to the model. An alternative possibility would be that listeners do not have knowledge of the distribution of f0 values, and the raw f0 values and the random effect of speakers mainly contributed to listeners’ judgement.

Therefore, in order to provide statistical evidence with a model comparison, we followed the procedure of comparing a set of models using AIC values considering log likelihood and number of estimable parameters proposed by Burnman and Anderson^21^. Among all the models in one set, the model with the least AIC value (AICmin) is identified, and the difference between each model and AICmin is calculated. If the difference is a value between 0 and 2, then the model has substantial empirical support. If the difference is between 4 and 7, then the level of empirical support is substantially less. If the difference is greater than 10, than the model can be omitted from further consideration since there is essentially no empirical support for the model^21^.

To further test whether the knowledge of f0 distributions or raw f0 values contributed to tonal judgment, we considered three models: a baseline model with only the random effect of speakers (model_0), a model with raw f0 values as the fixed effect and the random effect of speakers (model_1), as well as our current model with three parameters as fixed effects and the random effect of speakers (model_2). The AIC value of model_0 is 8766, model_1 is 8131, and model_2 is 7876. Among all three models, our current model (model_2) has the smallest AIC value (AICmin) 7876. We then calculated the difference between the AIC of each model and the minimum AIC value. The difference between the AIC of model_2 and AICmin is 0, the difference between the AIC of model_1 and AICmin is 255, and the difference between the AIC model_0 and AICmin is 890. Therefore, our current model has substantial empirical support, and the other two models are not supported by statistical evidence.

In addition, LOESS (locally weighted scatterplot smoothing) curves were fitted to the identification rate of T3 against each parameter (location, scale, shape) for the native listeners. The curves were fitted to examine how identification rate changes with respect to the value changes of each parameter. These identification curves can reflect if listeners have the ability to further calculate the probability of extracted parameters from tonal distributions of multiple speakers. If they have this ability, then their identification curves should change as the parameter values change. Note that the LOESS models were fitted to the identification rate of T3, not the raw data of discrimination results (Tone 1, Tone 3 or Tone 6). The model serves as a curve fitting technique to show how identification curve changes with respect to each parameter. It aims to examine whether as the values of each parameter (location, scale, shape) changes, the identification rate will increase or not. By flat LOESS curves, we mean that the curve is close to a straight horizontal line, which means that as the parameter changes, there is no change in the identification rate because the value of the identification rate remains to be the same. In other words, a flat curve means that the parameter has little influence on changing the identification rate.

**Figure 3:**
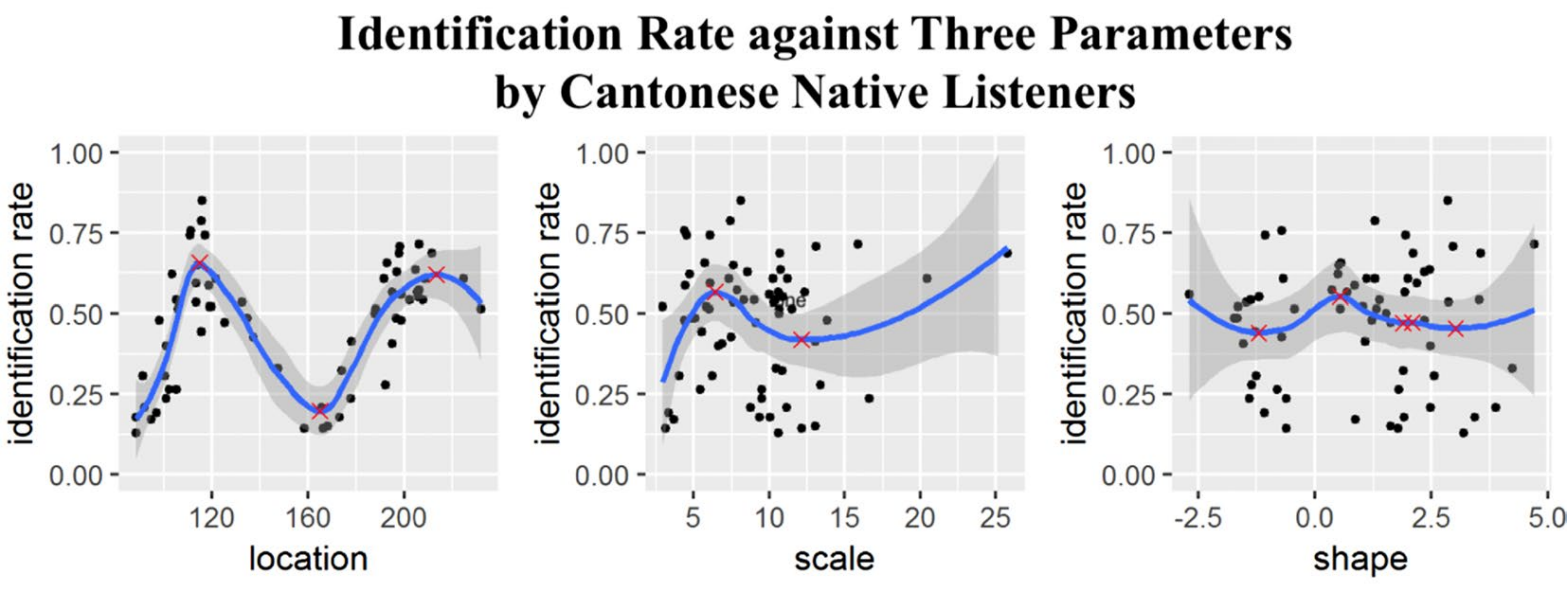
The graphs of the identification rate against the location, scale, and shape by native listeners.

The curves may also show peaks, which are considered as the most typical parameter of distributions from multiple speakers. If listeners do not have this ability, then identification curves will be relatively flat no matter how the values of each parameter change. In Fig. 3, the blue curves are the LOESS curves, and the grey regions are 95% confidence interval. The red cross signs refer to the local maximum or local minimum points. From Fig. 3, we can infer that the graphs of the identification rate against the location have two peaks (at location = 115 Hz and 213 Hz for native listeners). The two peaks in the two figures of location correspond to the location values that are most likely to be identified as T3 spoken by male and female speakers. The identification rate increased if the location parameter approached the two peaks and it decreased once the location parameter was away from the two peaks. The identification curves thus suggested that listeners were highly sensitive to the change of location parameter and they effectively represented location of f0 distributions for both male and female speakers.

Moreover, the identification rate reached its maximum point when the scale parameter ${\upomega }$ was at around ${\upomega } = 6$.38 in the interval $0 \le {\upomega } \le 15$. Note that the scale parameter represents the dispersion of f0 values. In the range of $0 \le {\upomega } \le 15$, the identification rate first increased and then deceased. The tonal identification also varied by the scale parameter. The estimation of f0 distributions from speech production suggested that speakers could not produce completely flat tones with one fixed f0 value (e.g. T1, T3, and T6), but produced f0 values with some variability. From the identification curve, native Cantonese speakers increased to the peak and then decreased in the identification rate as the scale parameter increased. A scale parameter either too small or too large would lead to a decrease in perception accuracy.

For the shape parameter α, Fig. 3 shows that native speakers demonstrated the highest identification rate at around α = 0.54 (which means the distribution is right skewed) and the identification rate decreased once the shape parameter was away from this peak. In sum, our calculations of all the maximum values of the curves showed that Cantonese listeners performed the best when the location ${\upxi }$ = 115.06 (male) or 213.34 (female), scale ${\upomega }$ = 6.38 and shape ${\upalpha }$ = 0.54.

Essentially, the mixed effects models capture the contribution of the speaker-dependent distributional characteristics to the probability of classifying the produced tone correctly. Considering the results from LOESS curves, the further the speaker-dependent distributional parameters for the given tone from the "the most typical parameter of distributions from multiple speakers" (corresponding to the "peaks" in Fig. 3), the more likely will the heard tone be mis-classified.

Moreover, in order to examine how well native speakers established mental representations of tonal distributions, we proceeded to compare the f0 distributions of each tone produced by the recruited 34 native speakers and the distribution of each tone identified by native listeners. A skew-normal distribution was used to fit the f0 values of the three tones (T1, T3 and T6) produced by 34 native Cantonese speakers recruited for speech recording of stimuli. We did not use the estimated distributions from the corpus in this analysis because we only used the corpus to estimate population f0 distributions of tones before conducting Experiment 2 for better manipulation of f0 values. In Experiment 1, the Cantonese listeners heard real stimuli produced by the newly recruited 34 native Cantonese speakers. Therefore, the f0 distributions produced by the 34 speakers and the f0 distributions based on the tones identified to be T1, T3 and T6 respectively in the experiment were more comparable.

**Figure 4:**
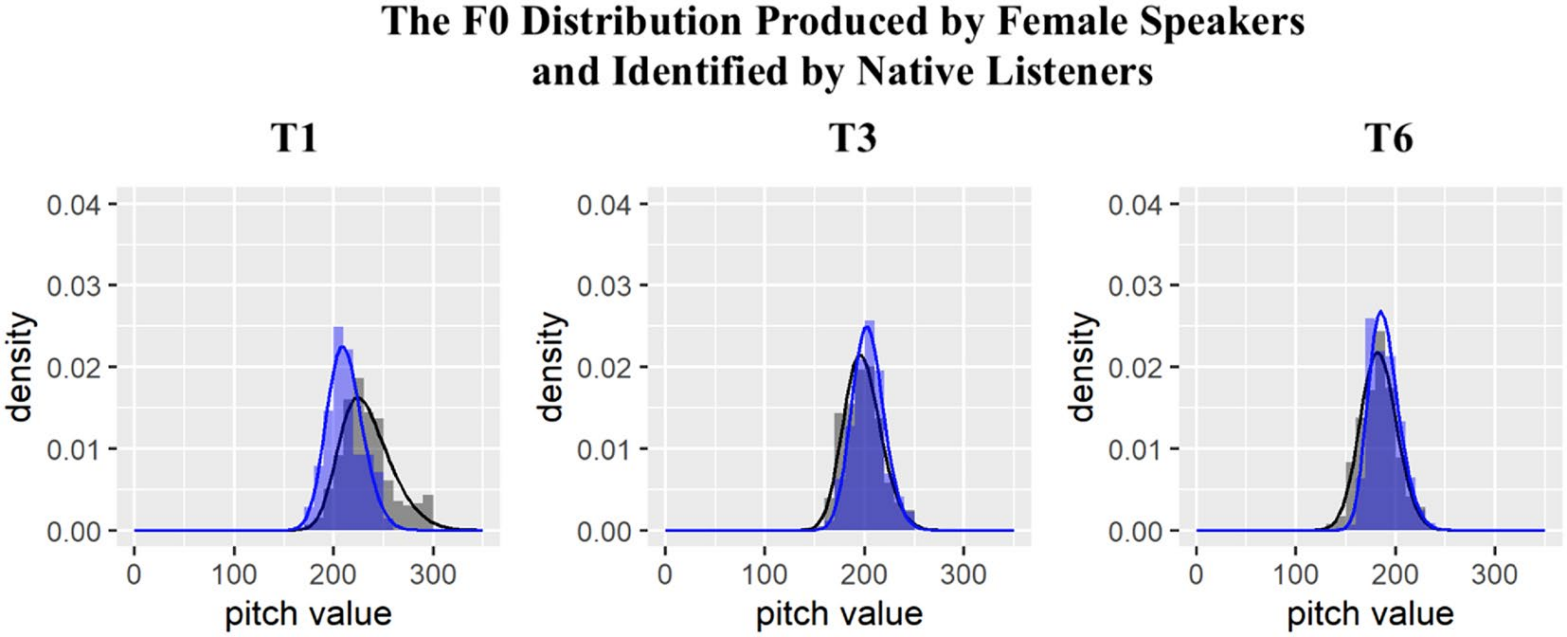
The f0 distribution of T1, T3, and T6 produced by female speakers (black color) and the f0 distribution of T1, T3, and T6 identified by native listeners (blue color).

**Figure 5:**
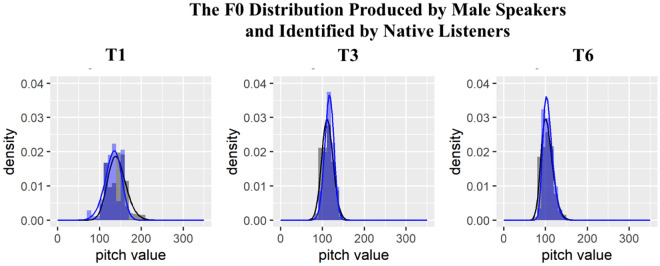
The f0 distribution of T1, T3, and T6 produced by male speakers (black color) and the f0 distribution of the response T1, T3, and T6 identified by native listeners (blue color).

Since we would like to test how native listeners establish tonal representations with respect to gender, we plotted the figures separately for male and female speakers. In Figs. 4 and 5, the black region and black line represent the frequency of f0 values and the estimated f0 density of T1, T3, and T6 produced by the male and female speakers respectively. The blue region and blue line refer to the frequency of f0 values and estimated f0 density of tones identified as T1, T3, and T6 by native Cantonese listeners. When plotting Figs. 4 and 5 and conducting the Kolmogorov–Smirnov test, we removed the outliers of f0 values following the Tukey outlier rule, which identifies any values more than below the first quartile or above the third quartile [69, page 61]. Figures 4 and 5 demonstrate a large overlapping area between the f0 distributions estimated from speech production and speech perception, indicating that listeners may represent f0 distributions in their long-term memory and recall it to achieve tone identification. However, Kolmogorov–Smirnov test showed that the f0 distribution of each tone produced by the speakers and the f0 distribution of each tone identified by the listeners were significantly different as shown in Table 1. An anonymous reviewer points out that the discrepancy found in the results may be due to the fact that the characteristics of the 34 speakers chosen in this study were relatively far from the characteristics of ideal speakers where the identification rate was much higher.

**Table 1:** The K-S statistic and p-value of the equality of the pitch distribution of each tone produced by Cantonese speakers and the pitch distribution of each tone identified by Cantonese listeners.

		K-S statistic	*p*-value
Cantonese listeners	T1 female speakers	0.42	< 0.001
	T3 female speakers	0.15	< 0.001
	T6 female speakers	0.17	< 0.001
Cantonese listeners	T1 male speakers	0.29	< 0.001
	T3 male speakers	0.18	< 0.001
	T6 male speakers	0.18	< 0.001

#### Discussion

Using LOESS (locally weighted scatterplot smoothing) curves to fit the identification rate against each parameter (location, scale, shape), we found that Cantonese listeners exhibited identification curves changing dramatically with respect to the three parameters of estimated speakers’ distributions in tone perception. Two peaks were found for the identification curve with respect to the location parameter and one peak was found for the curve with respect to the scale and shape parameter respectively. These results thus indicate that listeners were sensitive to the change of the parameters of tonal distributions and they effectively represented the location of f0 distributions for both male and female speakers. In addition to the sensitivity of parameters in speech perception, the results also suggested that native speakers were able to calculate the probability of parameters of individual speakers’ distributions to some extent using their knowledge about phonetic distributions of speakers in the Hong Kong Cantonese Speech Community. This step of processing can be viewed as a step to form a more “abstract” representation, integrating knowledge from previously established phonetic distributions.

The representation can be considered as a PPR, where not only can the parameters be updated with more exposure^31^, the probability of each parameter can also be updated with exposure to more speakers. The abstract representation can be generalized, which helps the processing of new forms. It is calculated relative to indexical information, which makes this representation go beyond the debate between episodic and abstract representations and furthers our understanding on parametric representations. This PPR can also be used to explain the possibility of co-existing levels of representations as in Zhang’s proposal^40^. The more abstract levels of representations such as parametric representations can be built up on the calculation of probabilities based on a lower level of representations with more phonetic details.

However, when we compared the distributions of tones in speech production and perception, the results showed that Cantonese speakers could not re-build the statistical distributions with no significant differences from those estimated from speech production. The distributions estimated from production and perception do overlap to a great extent. The observed results are probably due to the fact that listeners only have interactions with a limited number of speakers from the speech community, which makes the establishment of the distributions different from estimated distributions of the speech community based on speech production data. It is thus likely that individuals may show estimation bias due to their own personal experience with multiple talkers. In order to further test how deviation from parameters of estimated population distribution impact tone perception, we designed Experiment 2 using re-synthesized stimuli.

### Experiment 2: tone identification in isolation with manipulated f0 values

#### Subjects

Fourteen Cantonese speakers (7 female, 7 male; mean age ± sd: 19.71 ± 1.27) took part in Experiment 2. Since the stimuli of Experiment 2 were manipulated f0 values based on real stimuli presented in Experiment 1, we did not use the same participants in Experiment 1 to avoid any potential influences from participating in Experiment 1. All participants were paid to participate in the experiments, and signed informed consent forms in compliance with a protocol approved by the Human Subjects Ethics Sub-committee at the Hong Kong Polytechnic University. All protocols are carried out in accordance with relevant guidelines and regulations. Participants who reported any history of speech, hearing, or language difficulties were not included.

#### Estimation of distribution parameters from a Cantonese corpus

We estimated the distribution of tones produced by 68 speakers (34F, 34 M) in a Cantonese speech corpus, Cantonese Sentences for Continuous Speech Recognition System (CUSENT) developed by the Department of Electronic Engineering at the Chinese University of Hong Kong^33^. Each speaker produced approximately 300 utterances, from which we chose 50 sentences, amounting to 3,400 utterances in total. We selected only complete clauses that included both subject and predicate, and prioritized sentences containing syllables carrying T1, T3, and T6. The vowel portion of each syllable was segmented and the Praat script ProsodyPro ^63^ was used to extract f0 values at 20 normalized time points for each segmented vowel. We found that a skew-normal distribution may best capture the tonal distributions, where simulated tonal distributions did not differ significantly from the original distributions as shown below. Therefore, we fitted a skew-normal distribution to data points extracted from the f0 trajectories for T1, T3, and T6, respectively.

Recall that the skew-normal distribution SN(ξ, ω^2^, α) has three parameters, the location ξ, scale ω and shape α. The location parameter determines the location of the distribution, the scale parameter determines the dispersion, and the shape parameter determines the skewness. For female speakers, T1 follows a skew-normal distribution SN(222.59, 46.85^2^, 1.59), T3 ~ SN(190.43, 35.38^2^, 1.44) and T6 ~ SN(181.77, 32.9^2^, 1.37). For male speakers, T1 follows a skew-normal distribution SN(116.06, 30.39^2^, 1.78), T3 ~ SN(101.86, 23.55^2^, 1.61) and T6 ~ SN(96.59, 21.89^2^, 1.72). Using the function “rsn” in the package SN^68^ in R, we proceeded to simulate the estimated skew-normal distributions to compare with the distributions of original data. Application of the Kolmogorov–Smirnov test suggested that the simulated data and the original data were from the same distribution for all tones—the p-values were all greater than the significance level (5%), hence we failed to reject the null hypotheses that they were from the same distribution (Female: T1: D = 0.02, p = 0.56, T3: D = 0.01, *p* = 0.84; T6: D = 0.02, *p* = 0.51; Male: T1: D = 0.02, *p* = 0.59; T3: D = 0.02, p = 0.09; T6: D = 0.02, *p* = 0.23).

#### Manipulated f0 values

The f0 values were manipulated according to simulated distributions designed to deviate from estimated population distributions. All the parameters of simulated distributions for female and male speakers are listed in Table 2. According to Table 2, we set the estimated population distribution of mid tone by female (T3 ~ SN(190.43, 35.38^2^, 1.44)) and male (T3 ~ SN(101.86, 23.55^2^, 1.61)) speakers as baselines, and vary each parameter (location, scale and shape) respectively, fixing other parameters. For example, the first column “Female Location” in Table 2 means that we kept the scale and shape parameter fixed to be 35.38 and 1.44 while changing the location parameter (from 181.77 to 222.59).

**Table 2:** Parameters of simulated distributions.

ParameterDistribution	Female Location ξ (ω = 35.38, α = 1.44)	Female Scale ω (ξ = 190.43, α = 1.44)	Female Shape α (ξ = 190.43, ω = 35.38)	Male Location ξ (ω = 23.55, α = 1.61)	Male Scale ω (ξ = 101.86, α = 1.61)	Male Shape α (ξ = 101.86, ω = 23.55)
1	181.77	32.9	− 1.59	96.59	21.89	− 1.78
2	186.1	34.14	− 1.44	99.23	22.72	− 1.61
3	190.43	35.38	0	101.86	23.55	0
4	198.47	41.12	1.44	105.41	26.97	1.61
5	206.51	46.85	1.59	108.96	30.39	1.78
6	214.55			112.51		
7	222.59			116.06		

We chose the values of the three parameters to match or deviate from those of the estimated T3 male and female speakers’ tonal distributions respectively. We ensured that enough data points were covered to study changes in identification accuracy with respect to deviation of parameters. For female speakers, the location parameters of T1 and T6 have a difference of 40.82 Hz, based on which we designed the values of location shift from the estimated T3 population distribution in order to cover enough data points. Specifically, we cut off the distance between T3 and T6, as well as the distance between T1 and T3 into even steps. Similarly, we cut off the distance of scale parameters between T1 and T3 and that between T3 and T6 into even steps. For the shape parameter, we were interested in the value 0 because the skew-normal becomes normal when the shape parameter is 0. Also, the negative values were included to investigate whether both the left and right-skewed distributions affect tone perception. Similar procedure was applied to manipulate male speakers’ parameters.

**Figure 6:**
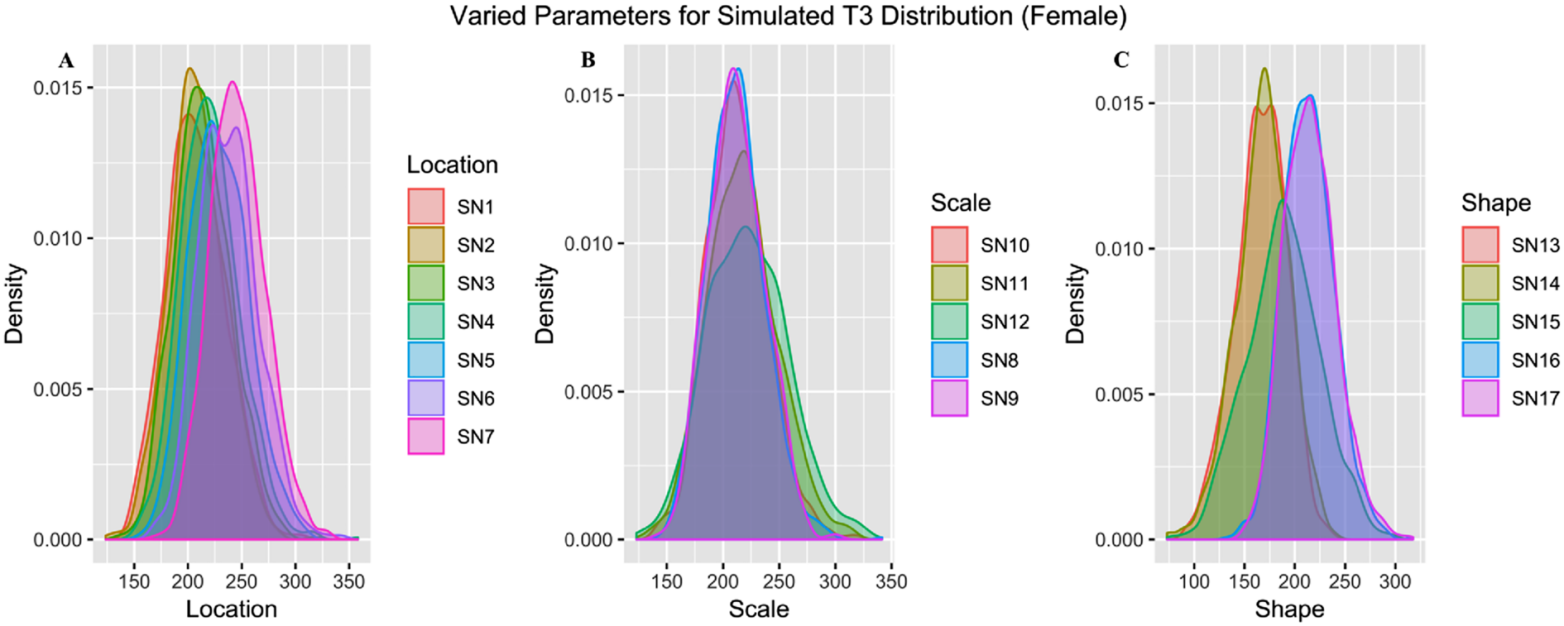
Density plot of simulated female skew-normal distributions with varied location (A), scale (**B**) and shape (**C**) parameters.

In total, we simulated 34 distributions based on collected speech of female and male speakers (17 female: 7 female location + 5 female scale + 5 female shape; 17 male: 7 male location + 5 male scale + 5 male shape). In Fig. 6, we show examples of the simulated distributions for different combinations of skew-normal parameter values. We randomly chose 10 data points from each simulated distribution for the identification task. In total, there were 340 (10 data points * 34 simulated distributions) target stimuli.

Each stimulus was manipulated as a level tone bearing a level pitch contour with the f0 values equal to the extracted data points from 34 distributions. We manipulated the pitch contour of each target syllable using the pitch synchronous overlap add (PSOLA) method^69^ implemented in Praat^64^. The target syllable was /ji/, and we also used a filler syllable /si/ in order to keep participants focused and interested on the task. The fillers were monosyllable /si/ bearing all of the same f0 values as the targets. The ratio of target words and fillers was 1:1, amounting to 680 stimuli in total.

#### Procedure

The procedure of Experiment 2 was similar to that of Experiment 1, except that the stimuli were synthesized stimuli with manipulated f0 values. The two sets of stimuli (target and filler stimuli) were presented in isolation using the three-alternative forced choice identification task. The target and filler words produced by 34 talkers were mixed randomly. All the stimuli were blocked by gender. The order of 34 talker-blocks was also randomized. All the participants wore a headset GMH C 8.100. Similar to Experiment 1, they were instructed to choose one monosyllable they heard from either a set of three target Cantonese words醫 (/ji55/ “a doctor”), 意 (/ji33/ “meaning”), and 二 (/ji22/ “two”), or the set of three filler Cantonese words 師 (/si55/ “a teacher”), 試 (/si33/ “to test”), and 事 (/si22/ “matter”), by pressing buttons on a computer keyboard. Traditional Chinese characters and symbols for tonal contours were given upon hearing each stimulus. They were also notified about the gender of each speaker.

#### Results

This experiment tests whether native Cantonese speakers’ tone perception is affected by deviation of parameters from the estimated f0 population distributions. The f0 values were manipulated according to simulated distributions, which have deviated parameters from those estimated from speech production in the corpus CUSENT.

When T6 served as a baseline, the main effects of location (T1 against T6: OR = 0.0057, *p* < 0.001; T3 against T6: OR = 0.037, *p* < 0.001), shape (T1 against T6: OR = 1.86e-198, *p* < 0.001; T3 against T6: OR = 4.94e + 125, *p* < 0.001) and scale (T1 against T6: OR = 1.45e-11, *p* < 0.001; T3 against T6: OR = 1.37e-07, *p* < 0.001) were positively related to the probability that the tone was identified as T1 or T3 against T6. All the two-way and three-way interaction terms reached significance. Similarly, in identifying T1 against T3, the main effect of location (OR = 0.11, *p* < 0.001), scale (OR = 1.70e-05, *p* < 0.001) and shape (OR = 8.46e-87, *p* < 0.001) were statistically significant in tone identification. All the two-way and three-way interaction terms reached significance. The results thus showed that Cantonese listeners were sensitive to the deviation of parameters from the estimated f0 population distributions.

#### Discussion

Experiment 1 and 2 showed that Cantonese speakers exploited distributional information in speech perception. Experiment 1 demonstrated that the values of most parameters contributed significantly to Cantonese speakers’ tone identification using real stimuli. Experiment 2 further proved that deviation of parameters from the estimated population distribution was also detected by native Cantonese listeners using synthesized stimuli. However, native Cantonese listeners could not establish exactly the same distributions from those estimated from speech production. Recall that in Experiment 1, we estimated the f0 distributions of tones produced by the recruited 34 native speakers, and that of each tone identified by native listeners. Since all the listeners listen to the tones produced by the recruited 34 Cantonese speakers, a skew-normal distribution was used to fit the f0 values of the three tones (T1, T3 and T6) produced by 34 recruited native Cantonese speakers recruited for speech recording of stimuli. The results from these two experiments thus showed parametric nature of the mentally stored representations, though the representations may vary from those estimated from speech production.

Recall that there is a debate between abstract and episodic representations of speech sounds^12,15^. Ideally, some theories that may integrate both abstract and episodic representations are needed to explain the current findings. The conceptual work proposed by Zhang argued that abstract and episodic representations may co-exist^40^. She hypothesized that there might be three levels of representations: the low level, the intermediate level and the high level of representations. The low level of representations may consist of talker-specific phonetic details, and the stored exemplars may decay over time and can be replaced by new exemplars. The intermediate level is a more abstract level with certain acoustic details and characteristics of typical talkers. The high level contains representations of phonological abstract categories, which are considered talker-invariant. In^40^, the relationship between the lowest level of exemplars and the intermediate level of population representations is not specified, nor is it clearly stated how new exemplars will continue to shape or update the intermediate level.

Another possible approach to go beyond the debate of abstract and episodic representations is the proposal of parametric representations. This proposal can successfully link the abstract and episodic representations through the concept of parametric representations, which explains how phonetic details may impact representations in memory^17^. It is believed that listeners may build up representations of phonetic distributions and gradually update the parameters of phonetic distributions with more exposure to the speech sounds. Moreover, previous studies indicate that the representations are quite flexible so that listeners may adjust to the talker’s indexical information such as age and gender. Hence, their parametric representations may reflect indexical information of speakers^4,5^. However, the proposed parametric representations may not be able to account for the results from our experiments in that there is a need for further extracting the acoustic parameters from individual distributions and calculating probabilities.

Using real speech, Experiment 1 showed that listeners were sensitive to the deviation of distribution parameters and the values of most parameters contributed significantly to Cantonese speakers’ tone identification.

In Experiment 2, listeners identified synthesized stimuli simulated from distributions with deviated parameters from those of the estimated population distribution. Similar to the results from Experiment 1, the three parameters also contributed significantly to tone perception of synthesized stimuli, and results proved that native speakers were aware of deviation of parameters from estimated population distributions of tones. These results in Experiment 1 and 2 thus indicated that mentally stored distributional information significantly contributed to speech perception by native Cantonese speakers.

Based on the stored exemplars from multiple speakers, further calculations can be achieved to both estimate the population phonetic distributions of linguistic units by speakers in a speech community and to build distributions of the extracted parameters (e.g. location, scale and shape) from multiple speakers to calculate the probability of those parameters. A relatively abstract representations such as PPR can be considered as a representation based on the calculation of stored phonetic distributions and distributions of parameters in making judgement of linguistic units from unknown speakers. Therefore, native speakers may store exemplars of tones, and they are also likely to have some knowledge about population phonetic distributions as well as distributions of parameters extracted from individual speakers’ distributions. Even some exemplars may decay over time, they still have access to their knowledge and estimation of a variety of distributions. Also, to create an even more abstract level of phonemes, speakers need to separate social and lexical influences and the level of abstractness is proposed to depend on the separability of these influences^31^. As the identification of tones highly depend on the probability of the parameters from phonetic distributions of talkers and gender can be a factor leading to rather distinct tonal distributions, the level of abstractness in phonemic tones might be lower than other types of phonemes, which remains to be tested by more experiments in the future.

### Experiment 3: tone identification in various contexts

#### Subjects

Fourteen Cantonese speakers (7 female, 7 male; mean age ± sd: 20.43 ± 1.02 years) were recruited to participate in Experiments 3. All participants were paid to participate in the experiments, and signed informed consent forms in compliance with a protocol approved by the Human Subjects Ethics Sub-committee at the Hong Kong Polytechnic University. All protocols were carried out in accordance with relevant guidelines and regulations. Participants who reported any history of speech, hearing, or language difficulties were not included.

#### Stimuli

Previous studies have shown that shifted tonal contexts may change perception of the target stimuli by native listeners. However, whether mentally stored tonal distributions may interact with contextual information has yet to be examined.

In order to examine whether there is an interaction between the two factors mentally stored tonal distributions and contextual information, the stimuli were designed for Experiment 3. The optimal range of T3 identification can be considered as a typical f0 range where a tone is identified as T3. Now we propose some extreme situations where only one of these two factors plays a dominant role. Consider a situation when the target mid tone stimulus is embedded in a context with only simulated high tones, which have f0 values higher than the target sound. The f0 distance between the simulated high tones and the target sounds was modelled after the distance between high and low tones in the population distribution. If stored distributional information strongly determines tone perception, then the mid tone should still be identified as T3 despite the presence of contextual information. However, if contextual information plays a stronger role, then the mid tone should be identified as a low tone.

In reality, it is likely that the two forces interact in determining tone perception. A similar argument applies if the context is manipulated to consist of only low tones. The contextual information in this experiment was manipulated by embedding stimuli into two carrier sentences: 1) all high tones T1(55): 聽聽 /tʰiŋ tʰiŋ/_________ ; “Listen to _________”; 2) all low tones T6(22): 就係/tʰsɐu hɐi/________; “This is just __________”.

#### Manipulated f0 values

If a random variable Y follows a skew-normal distribution SN (ξ, ω^2^, α), then the expectation (mean) and variance of Y are as follows^70^:

2 $$E\left( Y \right) = \upxi + \upomega \sqrt {\frac{2}{\pi }} \frac{\upalpha }{{\sqrt {1 + \alpha^{2} } }}$$

3 $$\text{Var}\left( \text{Y} \right) = \upomega^{2} \left\{ {1 - \frac{{2\upalpha^{2} }}{{\pi \left( {1 + \upalpha^{2} } \right)}}} \right\}$$

We first calculated the distance between mean f0 values of T1 and T3 as well as T3 and T6, using estimated population parameters. To measure the distance between two mean f0 values, we use semitones as in the following equation:

4 $${\text{Number of semitones}} = \frac{12}{{\log_{10} 2}}*\log_{10} \left( {F_{02} /F_{01} } \right)$$

where *f0*_*1*_ is the lower f0, and *f0*_*2*_ represents the higher f0 ^71^. For female speakers, the estimated population mean of T1 is 254.23 Hz, T3 is 213.62 Hz and T6 is 202.97 Hz. There are 3.01 semitone differences between mean T1 and T3, 0.88 semitone differences between T3 and T6, and 3.90 semitone differences between T1 and T6. For male speakers, the estimated population mean of T1 is 137.20 Hz, the mean of T3 is 117.82 Hz and the mean of T6 is 111.69 Hz. There are 2.64 semitone differences between mean T1 and T3, and 0.93 semitone differences between T3 and T6, and 3.56 semitone differences between T1 and T6.

Based on the estimated distance between T1 and T6, we simulated the carrier sentences containing T1 and T6. First, f0 values of T3 stimuli were the same as the stimuli in Experiment 1. We manipulated the pitch contour of syllables using the pitch synchronous overlap add (PSOLA) method^69^ implemented in Praat^64^. T1 in carrier sentences “聽聽 /tʰiŋ tʰiŋ/_________ ;Listen to ______” containing high tones were simulated so that each f0 value of T1 was 3.90st (estimated distance between T1 and T6 based on the corpus of female speakers) higher than that of simulated T3 for female speakers, and 3.56st higher (estimated distance between T1 and T6 based on the corpus of male speakers) than that of simulated T3 for male speakers. Similarly, T6 in carrier sentences “就係/tʰsɐu hɐi/ ________; This is _____” were simulated so that each f0 value of T6 was 3.90st lower than that of simulated T3 for female speakers, and 3.56st lower than that of simulated T3 for male speakers.

**Figure 7:**
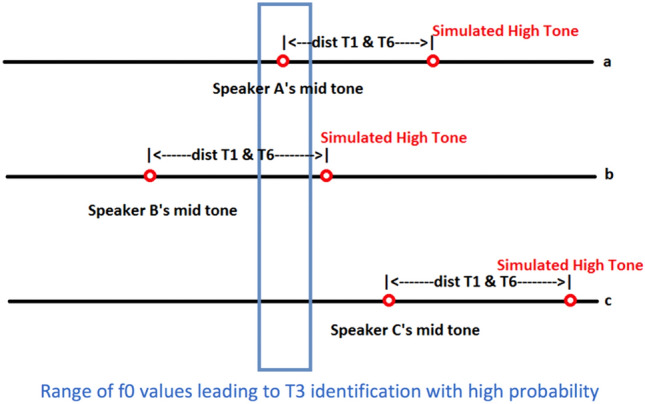
Simulated high tones (T1) treating mid tones (T3) as low tones (T6).

As shown in Fig. 7a, if the simulated f0 values representing a speaker’s mid tone T3 falls in a f0 range identified with high accuracy in isolation (optimal range henceforth), then the f0 values from that range came from tonal distributions of T3 with high probability. We may calculate the optimal range based on Experiment 1, where a tone is identified with the highest probability as T3 presented in isolation. The stored information of tonal distributions makes listeners tend to perceive a stimulus as T3. On the other hand, we simulate a high f0 context, which would lead to the identification of this T3 as T6 if the listeners used more contextual information to judge the target tone. In Fig. 7b, where the simulated f0 values representing a speaker’s mid tone T3 falls in a lower range than the optimal range, both stored distributional information and contextual information may cause the listeners to perceive it as a low tone T6. In Fig. 7c, the simulated f0 values representing a speaker’s mid tone T3 falls in a higher range than the optimal range. Therefore, stored distributional information may make the listeners perceive it as a high tone T1, but contextual information causes them to perceive it as a low tone T6.

**Figure 8:**
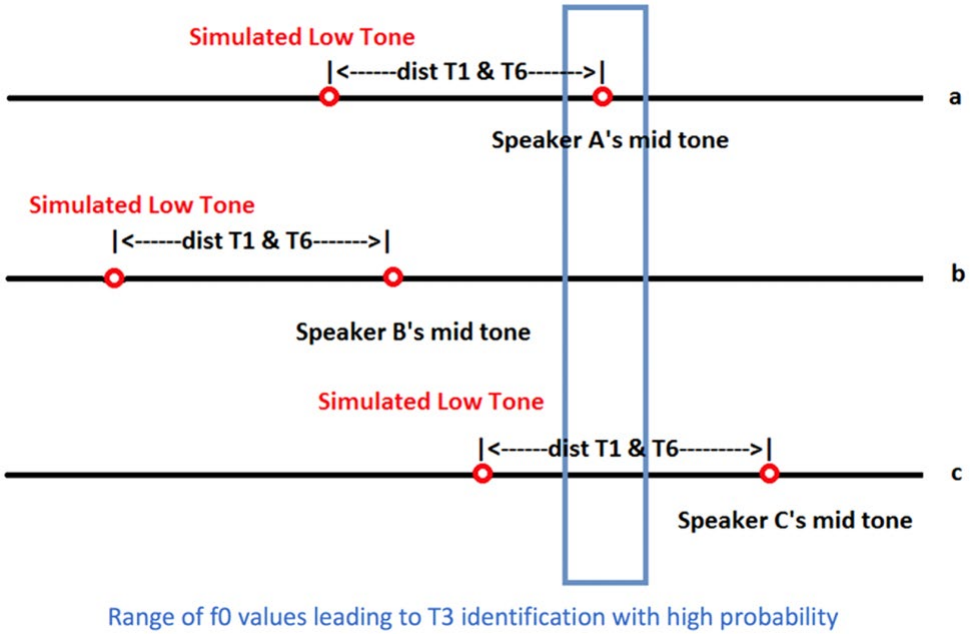
Simulated low tones (T6) treating mid tones (T3) as high tones (T1).

Similarly, in Fig. 8a, if the simulated f0 values of T3 falls in the optimal range, then listeners tend to perceive it as T3. However, the simulated low f0 context may cause listeners to perceive it as T1. In Fig. 8b, the simulated mid tone T3 falls in a lower range than the optimal range. Therefore, stored distributional information may cause the listeners to perceive it as a low tone T6, but contextual information may cause them to perceive it as a high tone T1. In Fig. 8c, the simulated mid tone T3 falls in a higher range than the optimal range. Both factors cause the listeners to perceive it as a high tone T1.

#### Results

Based on data obtained in Experiment 1, we calculated optimal f0 ranges for a tone to be identified as T3. The ranges were calculated so that the pitch values falling into the ranges were identified as T3 with the highest identification rate. The optimal pitch range for the female voice is the range [170.11 Hz, 238.21 Hz]. The optimal pitch range for the male voice is set to range [88.83 Hz, 134.16 Hz]. In order to measure the effect of the optimal pitch range and to test whether there is an interaction between stored distributional information and contextual information, an indicator function was introduced. For the f0 values presented in embedded sentences, the indicator function equals to 1 if the f0 value of the target word lies within the optimal pitch range, otherwise, it equals to 0. Significance of this indicator function means significant main effect from stored distributional information.

We proceeded to use data from Experiment 1 and 3 to fit multinomial mixed effects models with the indicator function, contextual effect and f0 values as independent variables while the tone identification as the dependent variable. Let $c$ be the tone level and ${y}_{ijk}$ be the tone perception of the $i$-th listener for the $j$-th speaker in the $k$-th replicate. The possible outcomes for ${y}_{ijk}$ were 1, 3, and 6. If we choose the possible outcome ${y}_{ijk}=6$ as a baseline and the other 2 outcomes (${y}_{ijk}$ = 1, 3) were separately regressed against the baseline outcome. The model is

5 $$P_{ijk6} = {\text{P}}\left( {y_{ijk} = 6{|}\beta_{i6} } \right) = \frac{1}{{1 + e^{{Z_{ijk1} }} + e^{{Z_{ijk3} }} }}$$

6 $$P_{ijkc} = {\text{P}}\left( {y_{ijk} = c{|}\beta_{ic} } \right) = \frac{{e^{{Z_{ijkc} }} }}{{1 + e^{{Z_{ijk1} }} + e^{{Z_{ijk3} }} }}$$

where $c = 1, 3$, $i = 1, 2, \dots , 14$, $j = 1, 2, \dots , 34$, and $k = 1, 2, \dots , 10$, ${Z}_{ijkc}={W}_{jk}{\gamma }_{c}+{\beta }_{ic}$, ${P}_{ijkc}$ is the probability that ${y}_{ijk}=c$ given ${\beta }_{ic}$, ${\gamma }_{c}$ is the parameters for fixed effects (the effect of intercept, f0 values, contextual effects, the indicator and the interaction terms), ${W}_{jk}$ is the observation of the $j$-th speaker with $k$-th replicate for fixed effects, and ${\beta }_{ic}$ is the parameter for random effects (i.e. the effect of the $i$-th listener). The p-value of each fixed effect is calculated by assuming that the fixed effects follow normal distributions.

Table 3 presents the results of the fitted model, which shows that the main effects of the indicator were statistically significant (for identification of T3 against T1 in simulated low tone contexts: OR = 1.32, *p* = 0.27; for identification of T3 against T1 in simulated high tone contexts: OR = 2.10, *p* < 0.001). These results indicate that for stimuli falling in the optimal range, it is more likely to be identified as T3 against T1). The contextual effects for both high tones (for identification of T3 against T1: OR = 3.78, *p* < 0.001) and low tones (for identification of T3 against T1: OR = 0.18, *p* < 0.001) were statistically significant. For most cases, contextual effects had a larger effect (based on higher absolute values of coefficients) on the identification of target tones than the indicator function, though the direction of identification rate change can be different in that the sign of the coefficients were different sometimes.

**Table 3:** Multinomial mixed effects model for simulated high tones “ting1 ting1 ji3” and simulated low tones “zau6 hai6 ji3” (T1 as the baseline).

	Simulated low tones			Simulated high tones	
	Estimate	*p*-value		Estimate	*p*-value
Intercept 3	186	< 0.001	Intercept 3	1.40	< 0.001
Intercept 6	3.05	< 0.001	Intercept 6	1.49	< 0.001
Pitch value 3	− 0.02	< 0.001	Pitch value 3	− 0.02	< 0.001
Pitch value 6	− 0.04	< 0.001	Pitch value 6	-0.03	< 0.001
Indicator 3	0.28	0.027	Indicator 3	0.74	< 0.001
Indicator 6	0.43	0.001	Indicator 6	1.99	< 0.001
tt 3	1.33	< 0.001	zh 3	− 1.73	< 0.001
tt 6	1.22	< 0.001	zh 6	− 3.78	< 0.001
Pitch*Indicator 3	0.02	< 0.001	Pitch*Indicator 3	0.01	< 0.001
Pitch*Indicator 6	0.02	< 0.001	Pitch*Indicator 6	0.01	< 0.001
Pitch*tt 3	0.02	< 0.001	Pitch*zh 3	0.01	< 0.001
Pitch*tt 6	0.03	< 0.001	Pitch*zh 6	0.02	< 0.001
Indicator*tt 3	− 0.25	0.174	Indicator*zh 3	− 2.39	< 0.001
Indicator*tt 6	− 1.40	< 0.001	Indicator*zh 6	− 3.28	< 0.001
Pitch*Indicator*tt 3	− 0.02	< 0.001	Pitch*Indicator*zh 3	0.00	0.48
Pitch*Indicator*tt 6	− 0.02	< 0.001	Pitch*Indicator*zh 6	− 0.01	0.048

Table 4 presents the results when T6 serves as the baseline. The main effects of the indicator function did not reach significance when the contexts were simulated low tones, but the main effects reached significance for high-tone contexts (for identification of T3 against T6: OR = 0.29, *p* < 0.001). In addition, the main effect of low-tone contextual information was not significant only for the identification of T3 against T6, but all of the main effect of the high-tone contextual information reached significance (for identification of T3 against T6: OR = 7.77, *p* < 0.001). Similar to Table 3, contextual effects had a larger effect (based on the absolute values of coefficients) on the identification of target tones than the indicator function for most cases.

**Table 4:** Multinomial mixed effects model for simulated high tones “ting1 ting1 ji3” and simulated low tones “zau6 hai6 ji3” (Tone 6 as the baseline).

	Simulated low tones			Simulated high tones	
	Estimate	*p*-value		Estimate	*p*-value
Intercept 1	− 3.05	< 0.001	Intercept 1	− 1.49	< 0.001
Intercept 3	− 1.19	< 0.001	Intercept 3	− 0.09	0.303
Pitch value 1	0.04	< 0.001	Pitch value 1	0.03	< 0.001
Pitch value 3	0.02	< 0.001	Pitch value 3	0.01	0.006
Indicator 1	− 0.43	0.173	Indicator 1	− 1.99	< 0.001
Indicator 3	− 0.15	0.148	Indicator 3	− 1.25	< 0.001
tt 1	− 1.22	< 0.001	zh 1	3.78	< 0.001
tt 3	0.11	0.314	zh 3	2.05	< 0.001
Pitch*Indicator 1	− 0.02	< 0.001	Pitch*Indicator 1	− 0.01	< 0.001
Pitch*Indicator 3	− 0.01	0.014	Pitch*Indicator 3	0.00	0.880
Pitch*tt 1	− 0.03	< 0.001	Pitch*zh 1	− 0.02	< 0.001
Pitch*tt 3	− 0.01	0.060	Pitch*zh 3	− 0.01	0.006
Indicator*tt 1	1.39	0.002	Indicator*zh 1	3.28	< 0.001
Indicator*tt 3	1.14	< 0.001	Indicator*zh 3	0.88	< 0.001
Pitch*Indicator*tt 1	0.02	< 0.001	Pitch*Indicator*zh 1	0.01	0.020
Pitch*Indicator*tt 3	0.00	0.861	Pitch*Indicator*zh 3	0.01	0.079

In sum, these results suggest that when the f0 values of a sound fall into the optimal pitch range, the probability that a listener perceives this sound as T3 increases for native Cantonese listeners in general. It is thus indicated that mentally stored distributional information still contributed to speech perception in the presence of contextual information in most cases. However, the contextual information generally contributed more to the identification of tones.

#### Discussion

The two factors of mentally stored long-term representations and the contextual effects of surrounding tones are seldomly considered together in speech perception experiments. Therefore, we designed Experiment 3 to test whether these two factors interact with each other in speech perception. Recall that we manipulated the surrounding tones in ways that the target tone would be perceived as a low or high tone, provided that contextual effects play a dominant role. On the other hand, if mentally-stored distributional information are to play a more dominant role in tone identification, then the target should still be perceived as a mid tone since the target tone is identified as a mid tone in isolation with high probability. Using an indicator function to test the interaction between the two forces, we found that both the main effects of contextual information and those of the indicator function reached significance in general. These results thus indicated that mentally stored distributional information still played a role when contextual information was present in speech perception, though the contributions might be smaller compared to that of contextual information in many cases.

These results suggest that speech perception is a more complex process than reported in previous studies, which integrates multiple factors including prior experience and knowledge about the talkers in a speech community, as well as immediate contextual information.

## General discussion

### Parametric representations and its competition with contextual information

The proposal of parametric representations shed light on the debate between abstract and episodic representations of speech sounds. This proposal goes beyond the abstract and episodic representations and explains how phonetic details may contribute to sound representations in memory^31^. Our experiments further explored mental representations of tones and tested the parametric nature of sound representations and how they are assessed during speech perception. There are two ways to test our hypothesis about the parametric nature of mental representations. We can either use natural stimuli from multiple speakers and extract parameters from each speaker (Experiment 1) or use synthesized stimuli (Experiment 2) based on values from simulated distributions with parameters deviated from the estimated population distribution. Using both ways of testing can offer us a fuller picture of what distributional information might be available to listeners.

Using real speech stimuli, Experiment 1 showed that listeners were sensitive to the deviation of distribution parameters and they were also able to represent the location parameters of f0 distributions for male and female speakers separately. In addition, native speakers calculated the probability of parameters extracted from individual speakers’ distributions, which is considered as a step to form a more “abstract” probabilistic parametric representation. Native speakers could mentally store distributional information and even calculate the probability of each parameter. The calculation may help them better integrate indexical information and make linguistic judgment. These findings agree with the idea of co-existing levels of representations proposed by^40^. Our results further revealed that it is possible for multiple levels of representations to exist, including the abstract levels of representations such as parametric representations or PPR, which are built up based on a lower level of representations with more phonetic details.

Experiment 2 further proved the parametric nature of sound representations using synthesized stimuli. As pointed by an anonymous reviewer, this experiment did not aim to imitate the natural situation as much as possible, but it follows the theoretical framework of underlying pitch targets for level tones. Xu and Wang proposed that a surface f0 contour is considered as the physical realization of the abstract linguistic unit of a lexical tone, which is subject to articulatory constraints in speech production^16^. Therefore, they proposed the concept of “underlying pitch targets” as “the smallest articulatorially operable units associated with linguistically functional pitch units” (p. 321). Chen et al. have shown that native speakers may categorize Mandarin pitch directions designed based on the underlying pitch targets estimated from Mandarin corpora^72,73^. Cantonese level tones have f0 values that do not vary much and under^74^’s theoretical framework, they have static level underlying pitch targets. Experiment 2 thus tested their identification of underlying pitch targets with no variation due to articulatory constraints. In this paper, we used a one dimensional distribution to model Cantonese level tones. In order to model the variations due to articulatory constraints or to model the slope of a rising tone, we will need to add a second dimension in the modelling of the distributions, e.g. a bivariate skew-normal distribution. This paper only tested PPR in the one dimensional case. For future studies, PPR can be further tested in multi-dimensional cases. These results in Experiment 1 and 2 thus indicated the contributions of mentally stored distributions in speech perception. Native speakers have knowledge about the population distributions of tones, and they have built up phonetic distributions for individual speakers and can have access to the probability of the parameters extracted from those distributions. Since parametric representations are at a more abstract level and will not be easily lost even when some exemplars of sounds in their memory fade away. The values and the probability of the parameters can also be updated with more exposure of speakers. For future studies, it is worth testing individual differences in building up sound representations and how the mental distributions are built by non-native learners in the process of sound acquisition and in what ways these differ from native listeners.

In addition to mentally stored long-term representations, evidence supporting the contribution of both category-intrinsic and category-extrinsic cues have been found in talker normalization^19,40^. Contextual information may largely influence listeners’ judgement of the incoming speech signals. For example, early studies showed that listeners may construct a talker-specific system incorporating information of other vowels produced by a certain talker in identifying incoming vowel signals^22^ and changes in the surrounding vowels may affect the target vowel identification^75^. Similarly, contextual information in surrounding tones has been found to affect tone identification of the target word^40,41,43,44^.

Experiment 3 was thus designed to test whether stored distributional information may still contribute to tone identification when contextual information was present. We used an indicator function to test the main effects of stored distributional information and contextual information. The results indicated that both of the main effects reached significance, suggesting that distributional information still played a role in speech perception even with the presence of contextual information.

The current model of talker normalization considers the processing of speech sounds produced by familiar and unfamiliar talkers as well as typical and less typical talkers. In that model, it is believed that if the talker characteristics of the incoming speech signal can be matched to those of a familiar talker, speech perception can be successful. If not, then typicality of the talker may largely determine the accuracy of speech perception since less typical talker lead to less accurate identification of linguistic units. Although unfamiliar and less typical talkers may be misidentified at first, contextual information may help with fast talker adaptation and the acoustical-to-phonological mapping for a particular talker^44^.

The findings from our experiments further revealed about the interactions between multiple factors in speech processing, especially in unfamiliar speakers. Specifically, our results indicated that in addition to native speakers’ capability of storing parametric representations of phonetic distributions and computing the probability of parameters, they kept having access to the computation capability and knowledge about phonetic distributions while processing contextual information. The current findings thus suggest a more integrated model of normalization is needed for the newly found evidence on the interactions between different sources of information. The degree of integration and the competition between these two sources of information in various situations still remains to be investigated. Since it is known that non-speech and other types of contexts also affect speech perception in ways similar to full speech contexts, though their effects may be reduced in some cases^44^, for future studies, it is worth exploring the interactions between long-term representations and various contextual effects in speech perception. It is likely that these factors may be integrated in different manners under various situations. It is worth exploring under what circumstances population distribution stops playing a role in perception and how the online processing works integrating conflicting information from the context effect and population distribution. It is possible that an initial percept is formed based on one source of info (e.g., population distribution), but this percept can be altered at a later stage integrating inform from other resources.

Results from these three experiments thus suggest that we need to proposal a model integrating multiple factors such as mentally stored distributional information, indexical information, as well as immediate contextual information. The results from our experiments revealed the integration of multiple factors in speech processing, especially in unfamiliar speakers. Native speakers were capable of building up parametric representations by updating parameters of phonetic distributions, computing the probability of parameters based on parameters extracted from individual speakers’ phonetic distribution with and without contextual information. Hence, we will need to update the model of talker normalization, which mainly focuses on speech processing of sounds by familiar vs. unfamiliar talkers as well as typical and less typical talkers^44^. The parameters of population distribution (location, scale and shape) and how the PPRs are updated via exemplars have not been specified in the previous model. The current study also manipulated contextual f0 based on modelled population distribution and tested whether the role of PPR and contextual information play.

### A new procedure integrating probabilistic parametric representations and contextual information

**Figure 9:**
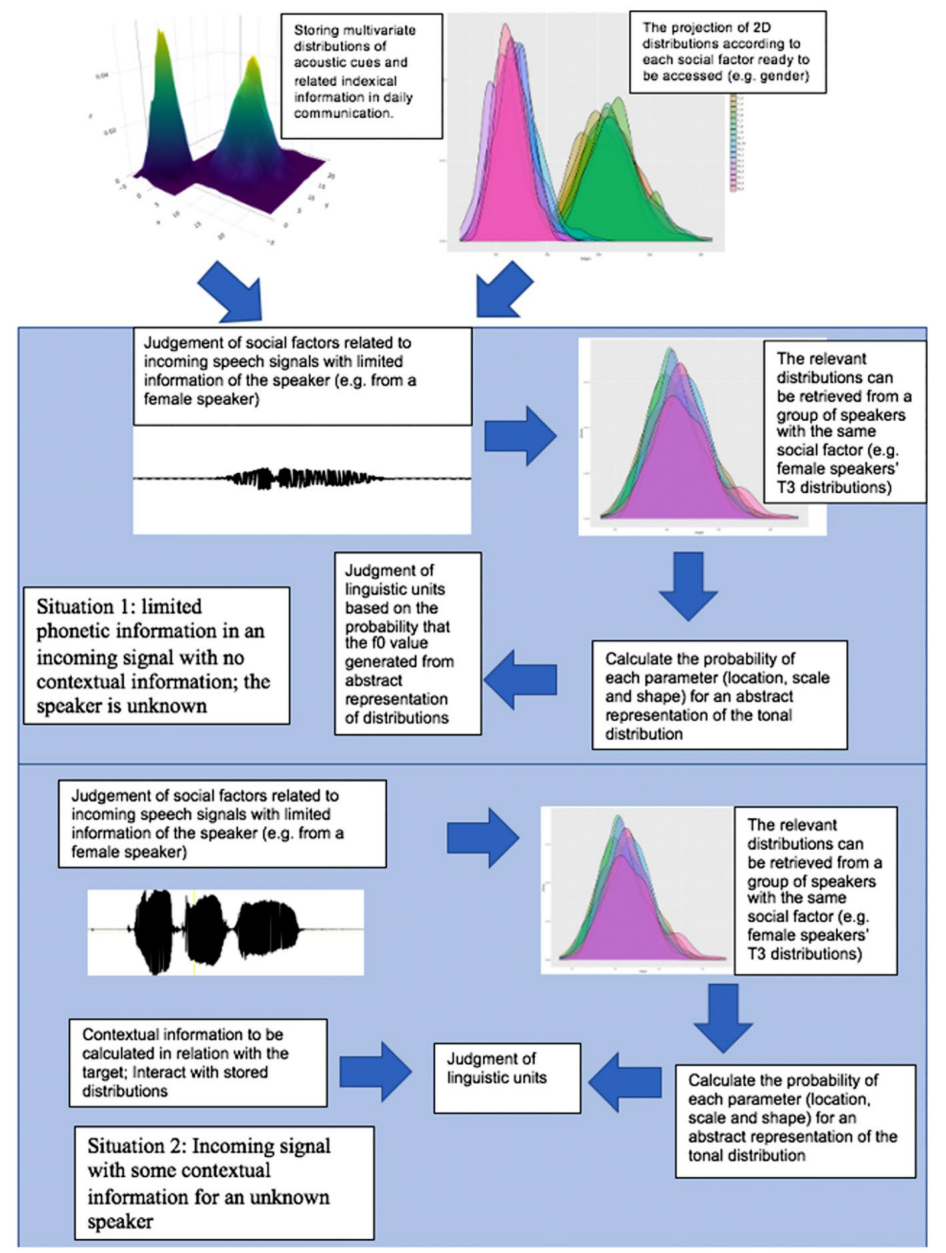
The hypothesized formation of parametric representations and assessment.

Based on findings in the literature^31,40,45^ and the results from the current study, we proposed a model of establishing and accessing mentally stored representations as shown in Fig. 9. In daily communication, a native listener builds up multivariate distributions of acoustic cues such as f0 values of speakers he or she interacted with, together with indexical information such as gender. A 3D plot of two bivariate distributions were provided as an example of high dimensional distributions. Kleinschmidt used quantitative methods to show that for a better speech perception result, listeners need to choose informative socio-indexical grouping factor such as gender in learning cue distributions^4^. Therefore, it is hypothesized that the stored multivariate distributions can be projected onto a two-dimensional space according to one factor of indexical information (e.g. gender) to be accessed during speech perception. For situation 1, where no contextual information is provided and the speech signal is from an unknown speaker, a listener will first be able to extract indexical information such as age and gender from the incoming speech signal^76^. The distributions relevant to the social factor (e.g. gender) and the judgment task (e.g. tones) can be retrieved from the mentally stored phonetic distributions of a group of speakers such as female speakers’ tone distributions. Second, based on the tonal distributions of multiple speakers, a native listener will further establish distributions of extracted parameters and he or she is able to calculate the probability of each parameter for each group of people (e.g. female speakers). Based on the results from Experiment 1, we may add this second step of forming a more “abstract” representation, namely a PPR, which may help listeners calculate probability of parameters when listening to a new speaker from previously established phonetic distributions. The established PPR is different from parametric representations since PPR is a representations of probabilities of parameters extracted from phonetic distributions of multiple speakers. The calculated probability can reflect the typicality of the phonetic distributions of a speaker. Finally, a native listener can make judgment of linguistic units such as tones based on the probability generated from the relative relation of the values of acoustic cues (e.g.f0 values) of incoming signals to the abstract representation of distributions. Similarly, if the incoming signal is from a known speaker, then the mentally-stored distributions of the speaker can be retrieved and the signal can be easily judged as shown in the model Computing Cues Relative to Expectations (C-CuRE), where acoustic cues were processed according to their differences from expected values. It is noted that the level of abstractness in the phonemic level may depend on the separability of social and indexical information^17^. Our results showed that the level of abstractness of phonemic Cantonese tones might be low since it highly depends on the distributional information of multiple speakers and indexical properties of speakers. Therefore, the procedure of storing phonetic distributions and distributions of parameters to form parametric representations and PPR is deemed to be indispensable for speech perception of Cantonese tones. It is reasonable to hypothesize that PPR and parametric representations may be less essential if social and indexical information can be easily separated and the phonemes are highly abstract. Thus, it remains to be tested whether PPR is necessary in normalizing for other types of linguistic units.

In situation 2, where some contextual information is provided for the speech signal from an unknown speaker, in addition to using the general auditory contrast enhancement mechanism^53^ for normalization, we found that native listeners still had access to the mentally stored distributions in making a linguistic judgment of the incoming speech signals. A native listener need to integrate information from mentally stored distributions and the contextual information from a new speaker in making a final decision. Although previous studies^44^ focused more on the normalization effect from various types of contextual information, our results showed a fuller picture of the role that information from stored distributions plays in the presence of contextual effects during speech perception. Since previous studies showed relatively consistent results regarding the significant contributions of contextual effects in Cantonese tone identification^43,44^, we may integrate previous findings and our findings in offering a fuller picture. It is noteworthy that the dominance relationship between the two forces can change in various conditions. The current results showed that contextual effects contributed more to tone identification than stored distributional information in most cases. The contextual effects can even play a more dominant role in certain conditions. For example, if contextual information contains enough acoustic cues for a listener to build up phonetic distributions of an unfamiliar speaker to make linguistic judgement, then the role of stored phonetic distributions may diminish significantly. On the other hand, if contextual information does not provide information about phonetic distributions of a speaker, stored phonetic distributions of other speakers may instead play a more important role and provide more information about potentially reasonable values of parameters. Future studies are called for to examine specifically about the interaction between these two forces for various types of contextual information including speech and nonspeech contexts.

## Conclusions

The current study examined the parametric nature of tonal representations and how mentally stored distributional information contributed in normalization of prosodic cues using real and synthesized stimuli. In addition, this study investigated whether stored distributional information may still play a role when contextual information is present. State of the art statistical techniques were applied and the results demonstrated significant contributions of stored distributions in speech perception of tones with and without contextual information. Based on these results, a model was proposed to explain the establishment of probabilistic parametric representations based on mentally stored distributions. The probabilistic parametric representation is different from previously proposed parametric representations in that this PPR provides distributional information about parameters extracted from distributions of speakers so that the probability of parameters can be calculated. Stored distributional information can be still be accessed when contextual information is present though it remains to be investigated whether contextual information may play a more dominant role than stored distributions in various contexts.

## Supplementary Information

Supplementary Information.

## Data Availability

Data are available.
