# Supplementary material for: Modelling representations in speech normalization of prosodic cues
Source: Sci Rep. 2022 Aug 27;12:14635. doi: 10.1038/s41598-022-18838-w (PMC9420126; doi:10.1038/s41598-022-18838-w)
Supplement: Supplementary file 1 — Supplementary Information. [file 41598_2022_18838_MOESM1_ESM.docx]

**Appendix**

The formulae for the multinomial mixed effects models are given below. Let $c$ be the tone level and $y_{ijk}$ be the tone perception of the $i$-th listener for the $j$-th speaker in the $k$-th replicate. The possible outcomes for $y_{ijk}$ were T1, T3, and T6. If we choose the possible outcome $y_{ijk}=T6$ as a baseline and the other two outcomes ($y_{ijk}$ = T1, T3) were separately regressed against the baseline outcome. The model is

$P_{ijk6}=P\left( y_{ijk}=6 | \beta_{i6} \right)=\frac{1}{1+e^{Z_{ij1}}+e^{Z_{ij3}}}$(1)

$P_{ijkc}=P\left( y_{ijk}=c | \beta_{ic} \right)=\frac{e^{Z_{ijc}}}{1+e^{Z_{ij1}}+e^{Z_{ij3}}}$ (2)

where $c = 1, 3$, $i = 1, 2, \ldots, 14$, $j = 1, 2, \ldots, 34$, and $k = 1, 2, \ldots, 10$, $Z_{ijkc}=W_{j}\gamma_{c}+\beta_{ic}$, $P_{ijkc}$ is the probability that $y_{ijk}=c$ given $\beta_{ic}$, $\gamma_{c}$ is the parameters for fixed effects (the effect of intercept, location, scale, shape, and all the interactions), $W_{j}$ is the observation of the $j$-th speaker for fixed effects (i.e. $\left[ \begin{matrix} 1 & \xi_{j} & \omega_{j} & \alpha_{j} & \xi_{j}\omega_{j} & \xi_{j}\alpha_{j} & \omega_{j}\alpha_{j} & \xi_{j}\omega_{j}\alpha_{j} \end{matrix} \right]^{T}$), and $\beta_{ic}$ is the parameter for random effects (i.e. the effect of the $i$-th listener).
